# Supplementary figures and images for: Horizontal transfer of probable chicken-pathogenicity chromosomal islands between Staphylococcus aureus and Staphylococcus agnetis
Source: PLoS One. 2023 Jul 5;18(7):e0283914. doi: 10.1371/journal.pone.0283914 (PMC10321648; doi:10.1371/journal.pone.0283914)

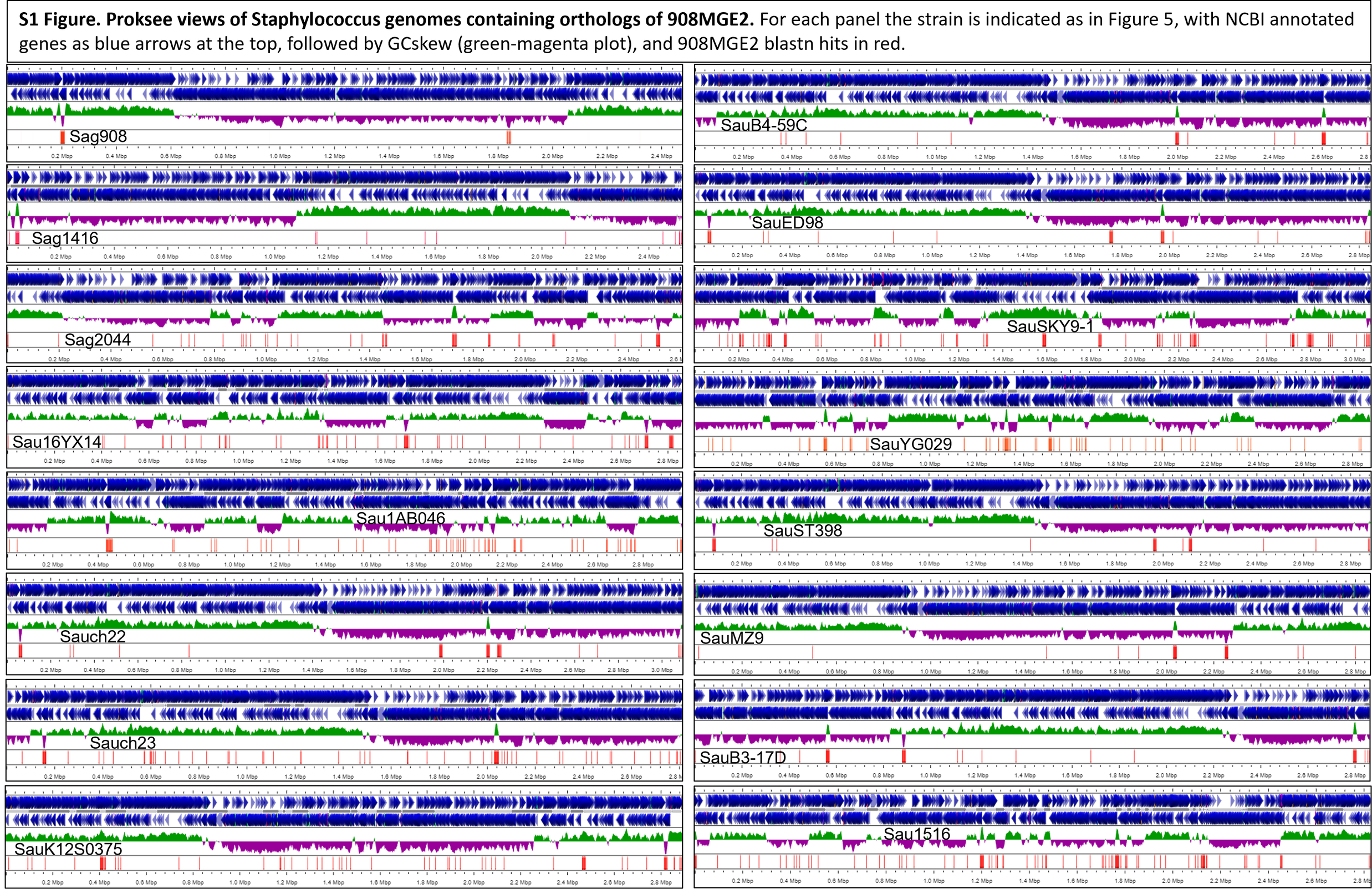

Supplement: S1 Fig — For each panel the strain is indicated as in Fig 5 with NCBI annotated genes as blue arrows at the top, followed by GCskew (green-magenta plot), and 908MGE2 blastn hits in red. (TIF) [file pone.0283914.s001.tif]
