# Supplementary material for: Horizontal transfer of probable chicken-pathogenicity chromosomal islands between Staphylococcus aureus and Staphylococcus agnetis
Source: PLoS One. 2023 Jul 5;18(7):e0283914. doi: 10.1371/journal.pone.0283914 (PMC10321648; doi:10.1371/journal.pone.0283914)
Supplement: S1 Table — For each genome the strain, species, and host/source, are based on the Biosample entry in NCBI. Genomes were sorted based on average percent identify for all 19 genes, and cells color coded (Green to Red) for percent identity using Microsoft Excel conditional formatting. (DOCX) [file pone.0283914.s002.docx]

**S1 Table. Percent Identity scores from a tBLASTn of *S. agnetis* and *S.aureus* genomes from NCBI when queried with PEG218-236 from the *S. agnetis* 908MGE2.** For each genome the strain, species, and host/source, are based on the Biosample entry in NCBI. Genomes were sorted based on average percent identify for all 19 genes, and cells color coded (Green to Red) for percent identity using Microsoft Excel conditional formatting.

| Strain | Species | Host/Source | 218 | 219 | 220 | 221 | 222 | 223 | 224 | 225 | 226 | 227 | 228 | 229 | 230 | 231 | 232 | 233 | 234 | 235 | 236 |
| --- | --- | --- | --- | --- | --- | --- | --- | --- | --- | --- | --- | --- | --- | --- | --- | --- | --- | --- | --- | --- | --- |
| 908 | *S. agnetis* | *Gallus gallus* | 100 | 100 | 100 | 100 | 100 | 100 | 100 | 100 | 100 | 100 | 100 | 100 | 100 | 100 | 100 | 100 | 100 | 100 | 100 |
| 1416 | *S. agnetis* | *G. gallus* | 100 | 100 | 100 | 100 | 100 | 100 | 100 | 100 | 100 | 100 | 100 | 100 | 100 | 100 | 100 | 100 | 100 | 100 | 100 |
| YG029 | *S. aureus* | *Sus scrofa* | 99 | 100 | 100 | 100 | 100 | 100 | 99 | 100 | 99 | 100 | 100 | 98 | 100 | 100 | 100 | 100 | 100 | 100 | 100 |
| 1AB046 | *S. aureus* | *Homo sapiens* | 99 | 100 | 100 | 100 | 100 | 100 | 99 | 100 | 99 | 100 | 100 | 98 | 100 | 100 | 100 | 100 | 100 | 100 | 100 |
| 16YX14 | *S. aureus* | pastry | 99 | 100 | 100 | 100 | 100 | 100 | 99 | 100 | 99 | 100 | 100 | 98 | 100 | 100 | 100 | 100 | 100 | 100 | 100 |
| ch22 | *S. aureus* | *G. gallus* | 99 | 96 | 100 | 100 | 100 | 100 | 100 | 100 | 99 | 100 | 100 | 100 | 100 | 100 | 100 | 100 | 100 | 100 | 100 |
| K12S0375 | *S. aureus* | chicken carcass | 100 | 100 | 100 | 100 | 100 | 100 | 98 | 98 | 99 | 99 | 99 | 100 | 100 | 100 | 100 | 100 | 100 | 100 | 100 |
| Chi-10 | *S. aureus* | *G. gallus* | 100 | 100 | 100 | 100 | 100 | 100 | 100 | 100 | 100 | 99 | 91 | 100 | 100 | 100 | 100 | 100 | 100 | 100 | 100 |
| ST398 | *S. aureus* | *H. sapiens* | 100 | 100 | 100 | 100 | 100 | 100 | 98 | 93 | 95 | 99 | 99 | 100 | 100 | 100 | 100 | 100 | 100 | 100 | 100 |
| 2011-60-2275-7 | *S. aureus* | *G. gallus* | 100 | 100 | 100 | 79 | 99 | 100 | 100 | 99 | 99 | 92 | 91 | 100 | 100 | 99 | 100 | 100 | 100 | 100 | 100 |
| 2009-60-561-1 | *S. aureus* | *G. gallus* | 100 | 100 | 99 | 99 | 100 | 100 | 100 | 99 | 99 | 99 | 0 | 98 | 99 | 99 | 100 | 100 | 100 | 100 | 99 |
| 2011-60-2275-1 | *S. aureus* | *G. gallus* | 100 | 100 | 0 | 100 | 100 | 100 | 98 | 93 | 95 | 100 | 0 | 100 | 100 | 100 | 100 | 100 | 100 | 99 | 82 |
| 22(2K81-5) | *S. aureus* | *G. gallus* | 100 | 100 | 0 | 100 | 96 | 100 | 98 | 93 | 95 | 99 | 0 | 100 | 100 | 100 | 100 | 100 | 100 | 99 | 82 |
| 09S00475 | *S. aureus* | *G. gallus* | 0 | 84 | 99 | 99 | 96 | 0 | 98 | 98 | 99 | 93 | 99 | 98 | 95 | 98 | 0 | 47 | 0 | 34 | 86 |
| MRSA-136 | *S. aureus* | *Meleagris gallopavo* | 0 | 84 | 99 | 99 | 96 | 0 | 98 | 98 | 99 | 92 | 99 | 98 | 95 | 98 | 0 | 49 | 0 | 34 | 82 |
| 15XX^a^ | *S. aureus* | *G. gallus* | 0 | 84 | 99 | 99 | 96 | 0 | 98 | 98 | 99 | 93 | 99 | 98 | 95 | 98 | 0 | 47 | 0 | 34 | 82 |
| N0426 | *S. aureus* | *Felis catus* | 0 | 84 | 99 | 99 | 96 | 0 | 98 | 98 | 99 | 93 | 99 | 98 | 95 | 99 | 0 | 0 | 0 | 34 | 82 |
| B3-17D | *S. aureus* | *G. gallus* | 0 | 84 | 99 | 99 | 96 | 0 | 98 | 98 | 99 | 93 | 99 | 98 | 95 | 98 | 0 | 0 | 0 | 34 | 82 |
| 5510 | *S. aureus* | *H. sapiens* | 0 | 84 | 99 | 99 | 96 | 0 | 98 | 98 | 99 | 93 | 99 | 98 | 95 | 98 | 0 | 0 | 0 | 34 | 82 |
| MSSA-123 | *S. aureus* | *M. gallopavo* | 0 | 84 | 99 | 99 | 96 | 0 | 98 | 98 | 99 | 93 | 99 | 98 | 95 | 98 | 0 | 0 | 0 | 34 | 82 |
| 2011-60-1490-31 | *S. aureus* | *G. gallus* | 0 | 84 | 99 | 99 | 96 | 0 | 98 | 98 | 99 | 93 | 99 | 98 | 95 | 98 | 0 | 0 | 0 | 34 | 82 |
| 37(18S2S5-05) | *S. aureus* | *G. gallus* | 0 | 84 | 99 | 99 | 96 | 0 | 98 | 98 | 99 | 93 | 99 | 98 | 95 | 98 | 0 | 0 | 0 | 34 | 82 |
| SA33924 | *S. aureus* | *H. sapiens* | 0 | 84 | 99 | 99 | 96 | 0 | 98 | 98 | 99 | 93 | 99 | 98 | 95 | 98 | 0 | 0 | 0 | 34 | 82 |
| RIVM1295 | *S. aureus* | *H. sapiens* | 0 | 84 | 99 | 99 | 96 | 0 | 98 | 98 | 99 | 92 | 99 | 98 | 95 | 98 | 0 | 0 | 0 | 34 | 82 |
| 25(2889) | *S. aureus* | *H. sapiens* | 0 | 84 | 99 | 99 | 96 | 0 | 98 | 98 | 99 | 92 | 99 | 98 | 95 | 98 | 0 | 0 | 0 | 34 | 82 |
| ED98 | *S. aureus* | *G. gallus* | 0 | 83 | 99 | 98 | 97 | 0 | 98 | 94 | 95 | 99 | 99 | 96 | 99 | 98 | 0 | 0 | 0 | 34 | 82 |
| R0487 | *S. aureus* | Rodentia | 0 | 83 | 99 | 99 | 96 | 0 | 97 | 97 | 96 | 93 | 99 | 99 | 97 | 99 | 0 | 0 | 0 | 34 | 81 |
| SNUC 1383 | *S. agnetis* | *Bos taurus* | 54 | 46 | 62 | 70 | 48 | 0 | 82 | 0 | 0 | 0 | 85 | 58 | 57 | 61 | 0 | 47 | 0 | 0 | 65 |
| 1392 | *S. agnetis* | *B. taurus* | 54 | 46 | 51 | 70 | 48 | 0 | 76 | 0 | 0 | 0 | 85 | 67 | 57 | 61 | 0 | 47 | 0 | 0 | 65 |
| SNUC 2265 | *S. agnetis* | *B. taurus* | 54 | 48 | 51 | 70 | 49 | 0 | 83 | 0 | 0 | 0 | 86 | 56 | 57 | 61 | 0 | 47 | 0 | 0 | 66 |
| 6 | *S. agnetis* | bovine | 54 | 46 | 51 | 70 | 48 | 0 | 76 | 0 | 0 | 0 | 86 | 58 | 57 | 61 | 0 | 48 | 0 | 0 | 65 |
| 59 | *S. agnetis* | bovine | 54 | 46 | 51 | 70 | 48 | 0 | 76 | 0 | 0 | 0 | 86 | 58 | 57 | 61 | 0 | 48 | 0 | 0 | 65 |
| 43 | *S. agnetis* | bovine | 54 | 46 | 51 | 70 | 48 | 0 | 76 | 0 | 0 | 0 | 86 | 58 | 57 | 61 | 0 | 47 | 0 | 0 | 65 |
| DSM 23656 | *S. agnetis* | Bovine | 54 | 46 | 51 | 70 | 48 | 0 | 76 | 0 | 0 | 0 | 85 | 58 | 57 | 61 | 0 | 48 | 0 | 0 | 65 |
| 722_230714_2_5_spleen | *S. agnetis* | *G. gallus* | 54 | 44 | 52 | 70 | 51 | 0 | 84 | 0 | 0 | 0 | 85 | 58 | 59 | 59 | 0 | 48 | 34 | 0 | 64 |
| 722_260714_1_8_heart | *S. agnetis* | *G. gallus* | 54 | 44 | 52 | 70 | 51 | 0 | 84 | 0 | 0 | 0 | 85 | 58 | 59 | 59 | 0 | 48 | 34 | 0 | 64 |
| 723_310714_2_2_spleen | *S. agnetis* | *G. gallus* | 54 | 44 | 52 | 70 | 51 | 0 | 84 | 0 | 0 | 0 | 85 | 58 | 59 | 59 | 0 | 48 | 34 | 0 | 64 |
| S69_POEL | *S. aureus* | *G. gallus* | 0 | 60 | 76 | 78 | 71 | 0 | 97 | 98 | 97 | 93 | 91 | 78 | 73 | 86 | 0 | 0 | 0 | 32 | 77 |
| 1387 | *S. agnetis* | *B. taurus* | 53 | 47 | 45 | 70 | 49 | 0 | 76 | 0 | 0 | 0 | 85 | 58 | 57 | 60 | 0 | 47 | 34 | 0 | 65 |
| NCTC9614 | *S. aureus* | avian | 98 | 58 | 75 | 79 | 70 | 0 | 94 | 70 | 0 | 0 | 91 | 78 | 73 | 86 | 0 | 0 | 0 | 31 | 85 |
| NCTC9613 | *S. aureus* | avian | 98 | 58 | 75 | 79 | 70 | 0 | 94 | 70 | 0 | 0 | 91 | 78 | 73 | 86 | 0 | 0 | 0 | 31 | 85 |
| NCTC9556 | *S. aureus* | *G. gallus* | 98 | 58 | 75 | 79 | 70 | 0 | 94 | 70 | 0 | 0 | 91 | 78 | 73 | 86 | 0 | 0 | 0 | 31 | 85 |
| pa3 | *S. aureus* | partridge | 98 | 58 | 75 | 78 | 70 | 0 | 94 | 70 | 0 | 0 | 91 | 78 | 73 | 86 | 0 | 0 | 0 | 31 | 85 |
| ch3 | *S. aureus* | *G. gallus* | 98 | 58 | 75 | 78 | 70 | 0 | 94 | 70 | 0 | 0 | 91 | 78 | 73 | 86 | 0 | 0 | 0 | 31 | 85 |
| ph2 | *S. aureus* | pheasant | 98 | 58 | 75 | 78 | 70 | 0 | 94 | 70 | 0 | 0 | 91 | 78 | 73 | 86 | 0 | 0 | 0 | 31 | 85 |
| ch5 | *S. aureus* | *G. gallus* | 98 | 58 | 75 | 78 | 70 | 0 | 94 | 70 | 0 | 0 | 91 | 78 | 73 | 86 | 0 | 0 | 0 | 31 | 85 |
| NCTC9546 | *S. aureus* | hen | 98 | 58 | 75 | 78 | 70 | 0 | 94 | 70 | 0 | 0 | 91 | 78 | 73 | 86 | 0 | 0 | 0 | 31 | 85 |
| NCTC9612 | *S. aureus* | avian | 98 | 58 | 75 | 78 | 70 | 0 | 94 | 70 | 0 | 0 | 91 | 78 | 73 | 86 | 0 | 0 | 0 | 31 | 85 |
| NCTC9611 | *S. aureus* | avian | 98 | 58 | 75 | 78 | 70 | 0 | 94 | 70 | 0 | 0 | 91 | 78 | 73 | 86 | 0 | 0 | 0 | 31 | 85 |
| UB489 | *S. aureus* | *H. sapiens* | 96 | 63 | 76 | 79 | 70 | 0 | 94 | 0 | 0 | 0 | 91 | 77 | 76 | 87 | 0 | 48 | 0 | 31 | 86 |
| c52 | *S. aureus* | *H. sapiens* | 96 | 63 | 76 | 79 | 69 | 0 | 94 | 0 | 0 | 0 | 91 | 77 | 74 | 88 | 0 | 48 | 0 | 31 | 86 |
| 1283_SAUR | *S. aureus* | *H. sapiens* | 98 | 62 | 76 | 79 | 70 | 0 | 94 | 0 | 0 | 0 | 91 | 78 | 73 | 87 | 0 | 48 | 0 | 31 | 86 |
| UP_1073 | *S. aureus* | *H. sapiens* | 97 | 63 | 75 | 79 | 70 | 0 | 94 | 0 | 0 | 0 | 91 | 78 | 74 | 88 | 0 | 0 | 0 | 32 | 86 |
| UB490 | *S. aureus* | *H. sapiens* | 96 | 63 | 76 | 79 | 70 | 0 | 94 | 0 | 0 | 0 | 91 | 77 | 76 | 87 | 0 | 0 | 0 | 31 | 86 |
| NCCP 16830 | *S. aureus* | *H. sapiens* | 97 | 62 | 76 | 79 | 70 | 0 | 94 | 0 | 0 | 0 | 91 | 77 | 74 | 88 | 0 | 0 | 0 | 31 | 86 |

1. 15XX represents assemblies of 1510, 1511, 1513 through 1524 which were determined to be clonal isolates from 7 lame birds in the same facility (Ekesi et al., 2021)
