# Supplementary material for: Horizontal transfer of probable chicken-pathogenicity chromosomal islands between Staphylococcus aureus and Staphylococcus agnetis
Source: PLoS One. 2023 Jul 5;18(7):e0283914. doi: 10.1371/journal.pone.0283914 (PMC10321648; doi:10.1371/journal.pone.0283914)
Supplement: Attachment — Submitted filename: Response to Reviewers.docx [file pone.0283914.s003.docx]

1. Please ensure that your manuscript meets PLOS ONE's style requirements, including those for file naming. The PLOS ONE style templates can be found at
   <https://journals.plos.org/plosone/s/file?id=wjVg/PLOSOne_formatting_sample_main_body.pdf> and 
   <https://journals.plos.org/plosone/s/file?id=ba62/PLOSOne_formatting_sample_title_authors_affiliations.pdf>

**Response: none required**

2. We note that the grant information you provided in the ‘Funding Information’ and ‘Financial Disclosure’ sections do not match.

When you resubmit, please ensure that you provide the correct grant numbers for the awards you received for your study in the ‘Funding Information’ section.

**Response: None of the funding for this project had grant numbers. They were all internal awards.**

3. In your Data Availability statement, you have not specified where the minimal data set underlying the results described in your manuscript can be found. PLOS defines a study's minimal data set as the underlying data used to reach the conclusions drawn in the manuscript and any additional data required to replicate the reported study findings in their entirety. All PLOS journals require that the minimal data set be made fully available. For more information about our data policy, please see <http://journals.plos.org/plosone/s/data-availability>.

"Upon re-submitting your revised manuscript, please upload your study’s minimal underlying data set as either Supporting Information files or to a stable, public repository and include the relevant URLs, DOIs, or accession numbers within your revised cover letter. For a list of acceptable repositories, please see <http://journals.plos.org/plosone/s/data-availability#loc-recommended-repositories>. Any potentially identifying patient information must be fully anonymized.

Important: If there are ethical or legal restrictions to sharing your data publicly, please explain these restrictions in detail. Please see our guidelines for more information on what we consider unacceptable restrictions to publicly sharing data: <http://journals.plos.org/plosone/s/data-availability#loc-unacceptable-data-access-restrictions>. Note that it is not acceptable for the authors to be the sole named individuals responsible for ensuring data access.

We will update your Data Availability statement to reflect the information you provide in your cover letter.

**Response: we apologize for not including the Supplementary Table 1 in our original submission. The table is now uploaded with the manuscript.**

4. We note that you have included the phrase “data not shown” in your manuscript. Unfortunately, this does not meet our data sharing requirements. PLOS does not permit references to inaccessible data. We require that authors provide all relevant data within the paper, Supporting Information files, or in an acceptable, public repository. Please add a citation to support this phrase or upload the data that corresponds with these findings to a stable repository (such as Figshare or Dryad) and provide and URLs, DOIs, or accession numbers that may be used to access these data. Or, if the data are not a core part of the research being presented in your study, we ask that you remove the phrase that refers to these data.

**Response: all occurrences of “data not shown” have been dealt with either removed or replaced with supplementary tables/figures.**

5. We note that you have referenced (unpublished) on page 22 which has currently not yet been accepted for publication. Please remove this from your References and amend this to state in the body of your manuscript: (ie “Bewick et al. [Unpublished]”) as detailed online in our guide for authors
<http://journals.plos.org/plosone/s/submission-guidelines#loc-reference-style>

**Response: The styles link documents what to do for “Accepted, unpublished articles”. At this time we have no plans to publish the information listed regarding S. agnetis 908 survival in macrophage-like cells, and it will likely remain “unpublished”. Further, many of the genomes from NCBI (Table 1) were submitted but never published. We await editor suggestions on Table 1, and have modified the text (lines 485-487)**

6. Please upload a copy of Supporting Information Figure/Table/etc. Supplementary Table 1 which you refer to in your text on page 9.

**Response: this has been done**

7. Please review your reference list to ensure that it is complete and correct. If you have cited papers that have been retracted, please include the rationale for doing so in the manuscript text, or remove these references and replace them with relevant current references. Any changes to the reference list should be mentioned in the rebuttal letter that accompanies your revised manuscript. If you need to cite a retracted article, indicate the article’s retracted status in the References list and also include a citation and full reference for the retraction notice.

**Response: not applicable**

[Note: HTML markup is below. Please do not edit.]

Reviewers' comments:

Reviewer's Responses to Questions

**Comments to the Author**

1. Is the manuscript technically sound, and do the data support the conclusions?

The manuscript must describe a technically sound piece of scientific research with data that supports the conclusions. Experiments must have been conducted rigorously, with appropriate controls, replication, and sample sizes. The conclusions must be drawn appropriately based on the data presented.

Reviewer #1: Yes

Reviewer #2: Yes

2. Has the statistical analysis been performed appropriately and rigorously?

Reviewer #1: Yes

Reviewer #2: Yes

3. Have the authors made all data underlying the findings in their manuscript fully available?

The [PLOS Data policy](https://nam11.safelinks.protection.outlook.com/?url=http%3A%2F%2Fwww.plosone.org%2Fstatic%2Fpolicies.action%23sharing&data=05%7C01%7Cdrhoads%40uark.edu%7C1f9a0b0748884a117d3708db5306b0eb%7C79c742c4e61c4fa5be89a3cb566a80d1%7C0%7C0%7C638195061508728467%7CUnknown%7CTWFpbGZsb3d8eyJWIjoiMC4wLjAwMDAiLCJQIjoiV2luMzIiLCJBTiI6Ik1haWwiLCJXVCI6Mn0%3D%7C3000%7C%7C%7C&sdata=vhoZgCWM9pyMunNgPdoTyUmul4y%2FrSt35rizTaJs%2Fro%3D&reserved=0) requires authors to make all data underlying the findings described in their manuscript fully available without restriction, with rare exception (please refer to the Data Availability Statement in the manuscript PDF file). The data should be provided as part of the manuscript or its supporting information, or deposited to a public repository. For example, in addition to summary statistics, the data points behind means, medians and variance measures should be available. If there are restrictions on publicly sharing data—e.g. participant privacy or use of data from a third party—those must be specified.

Reviewer #1: No

Reviewer #2: No

4. Is the manuscript presented in an intelligible fashion and written in standard English?

PLOS ONE does not copyedit accepted manuscripts, so the language in submitted articles must be clear, correct, and unambiguous. Any typographical or grammatical errors should be corrected at revision, so please note any specific errors here.

Reviewer #1: Yes

Reviewer #2: Yes

5. Review Comments to the Author

Please use the space provided to explain your answers to the questions above. You may also include additional comments for the author, including concerns about dual publication, research ethics, or publication ethics. (Please upload your review as an attachment if it exceeds 20,000 characters)

Reviewer #1: The article reports the discovery of a mobile genetic element (MGE) in Staphylococcus agnetis isolates from chicken osteomyelitis and dermatitis.This MGE is only found in chicken infections and can have multiple copies per genome.
It was vectored on a Staphylococcus phage and is related to intact MGEs found in S. aureus genomes.
According to the article, these MGEs may represent a new family of Chromosomal Islands shared by S. agnetis and S. aureus, and more research is needed to understand their role in pathogenesis.
I have a couple of minor remarks:
1-It would be great if the authors could include the species names in Figures 1–4 as they did in Figure 5.
Without the species names, there are too many strain names, which can be confusing.

**Response: Only Figure 5 includes multiple isolates of both strains so we included the genus-species abbreviated prefix there for the phylogenomic tree clarity. In the other figures the species are clearly defined in the legend, especially in figure 4 where only S. aureus isolates are in the tree. In figures 1 the legend states that it is only S. agnetis in the figure, Figure 2 is only two isolates of S. agnetis (made clearer in the revision), and in Figure 3 we clarified the species in the legend. Although the reviewers point is well taken that it is likely difficult to most to keep all the “players” clear. We have modified the manuscript to repeatedly specify the species so that it is easier to follow which species each isolate is.**

2-In Fig 2, at line 188, the authors mention dark green nodes.
Did they mean dark blue nodes, or are they referring to fig 3?

**Response: This is definitely our error in tracking figures during revisions. This should refer to the poppunk tree in our submitted Figure 4, and the nodes we are referring to are actually in red (as we rendered the tree through multiple iterations). To correct our mistake we switched Figure 3 and 4 in the revision. Figure citations have therefore been updated.**

3-From time to time, authors discuss data that lack figures (data not shown). Because these data complete the picture, I believe some of these data should be included in the supplementary figures.

**Response: We have added Figure 6 and S Figure 1 to provide additional data and remove the “data not shown”**

I am excited to learn more about how these MGEs contribute to pathogenesis.

Reviewer #2: 1. Manuscript switches between referring to an isolate with their numerical name e.g. line 101 and isolate "numerical name" e.g. line 107. Consistency is recommended. It is easier to follow the manuscript when isolates are referred as "isolate numerical name".

**Response: we have endeavored to do so throughout. We have modified the manuscript to repeatedly specify the species so that it is easier to follow which species each isolate is.**

2. Lines 107 and 109 refer to genomes as "most complete" but lack descriptive numbers to determine a genome as close to complete.

**Response: we have added the criteria for our selections. (lines 107-111)**

3. Line 111 verbiage "from 0.16 to just beyond 0.2 Mbp" is vague. Suggestion for replacement: " in the region between 0.16 - 0.24 Mbp ".

**Response: we have done so. (lines 112-114)**

4. Lines 131 and 245-246 mention insertion site for the MGEs. What are the nucleotide sequences of the insertion sites in the genomes mentioned? What are the bp numbers for the insertion site in the isolate 908 genome? With the lack of insertion site sequence, there is not enough evidence provided to support the claim that the insertion sites are distinct. Authors have compared annotations of the regions around the MGE but not supported that with nucleotide level identities of the surrounding regions.

**Response: we had not tried to identify the specific termini of the MGE because to know the insertion site you must have the prophage with and without the MGE. We had looked for direct repeats but did not describe this. Based on the reviewers comments we have reanalyzed genomes where there are the best candidates for sequences before and after insertion which has led to inclusion of Figure 6 where we analyzed the insertion site in S. agnetis for 908CI2 because we have a closely related cattle-isolate 1379, and then in S. aureus where ED98 has 3 insertions but the the closely related isolate B4-59C only has 2, so we can align the genomes for both these cases to specifically identify the island termini. We have therefore expanded the discussion about insertion site, and included Figure 6. We thank the reviewer for the comments as the new data and figure provide necessary details.**

5. What are the direct or indirect repeats associated with 908 MGE1, 908MGE2/CI1 and 908CI2?

**Response: see preceding response**

6. PCR is needed to the presence of the MGEs at the insertion region as determined by the genome assembly is correct.

**Response: it is not clear what the reviewer is requesting. Are they requesting that we verify that the assembly is correct through some sort of PCR test? Given that the focus is really on a family of chromosomal islands in multiple genomes and many of those genomes were assembled by others we could only have access to DNA from a few genomes.**

7. Line 165 mentions use of genome analyses service at BV-BRC to support all three duck isolates being S. aureus. Manuscript lacks the parameters used to conclude duck isolates as S. aureus.

**Response: we have included the specific metric (lines 167-169)**

8. Supplementary Table 1 mentioned in line 171 is missing.

**Response: We deeply apologize for this error on our part. The table has now been included**

9. Fig 2 referred in line 187 does not sow a phylogenetic tree. Did the authors mean Fig 4?

**Response: This error has been corrected and includes the switching of Figure 3 and 4.**

10. Data referred to in lines 219 - 222 should be included as supplementary figure.

**Response: We have done so.**

11. Table 2 legend: sentence beginning with "PEG is protein..." does not need to be bold.

**Response: corrected.**

12. Lines 281-286 need to be supported by data. Data can be added to the supplementary materials.

**Response: we have done so with S1 Figure.**

13. Line 303: should be a period instead of comma after "SauED98CI3".

**Response: Corrected (line 307)**
